# Synthesis, spectral analysis, quantum studies, NLO, and thermodynamic properties of the novel 5-(6-hydroxy-4-methoxy-1-benzofuran-5-ylcarbonyl)-6-amino-3-methyl-1*H*-pyrazolo[3,4-*b*] pyridine (HMBPP)

**DOI:** 10.1039/d2ra01469f

**Published:** 2022-04-29

**Authors:** Shimaa Abdel Halim, Magdy A. Ibrahim

**Affiliations:** Department of Chemistry, Faculty of Education, Ain Shams University Roxy 11711 Cairo Egypt shimaaquantum@ymail.com +20 1090306455

## Abstract

Ring opening followed by ring closure reactions of 4-methoxy-5-oxo-5*H*-furo[3,2-*g*] chromene-6-carbonitrile (1) with 5-amino-3-methyl-1*H*-pyrazole (2) afforded the novel 5-(6-hydroxy-4-methoxy-1-benzofuran-5-ylcarbonyl)-6-amino-3-methyl-1*H*-pyrazolo[3,4-*b*] pyridine (3, HMBPP). The chemical structure of the synthesized compound was established based on elemental analysis and spectral data. The chemical calculations were performed using the Becke3–Lee–Yang–Parr (B3LYP) and Coulomb Attenuating Method (CAM-B3LYP)/6-311++G(d,p) basis sets at the DFT level of theory. The Coulomb-attenuating method (CAM-B3LYP) and Corrected Linear Response Polarizable Continuum Model (CLR) PCM were used to obtain the theoretical electronic absorption spectra in the gas phase, methanol, and cyclohexane, respectively, indicating good agreement with the observed spectra. The local reactivity descriptors supported the high reactivity of C7 for nucleophilic attack. The computed total energy and thermodynamic parameters at the same level of calculations confirmed the high stability of structure 3 (HMBPP) as compared with the other expected structure 4. The ^1^H and ^13^C chemical shift values, as well as vibrational wavenumber values, were theoretically determined and exhibited a high correlation with the experimental data. Natural bond orbital analysis (NBO) was used to investigate hyper conjugative interactions. The first static hyperpolarizability, second hyperpolarizability, polarizability, and electric dipole moment have been determined. At different temperatures, the thermodynamic properties of the compounds were calculated.

## Abstract 2

Ring opening followed by ring closure reactions of 4-methoxy-5-oxo-5*H*-furo[3,2-*g*] chromene-6-carbonitrile with 5-amino-3-methyl-1*H*-pyrazole gave the novel 5-(6-hydroxy-4-methoxy-1-benzofuran-5-ylcarbonyl)-6-amino-3-methyl-1*H*-pyrazolo[3,4-*b*] pyridine.

## Graphical abstract

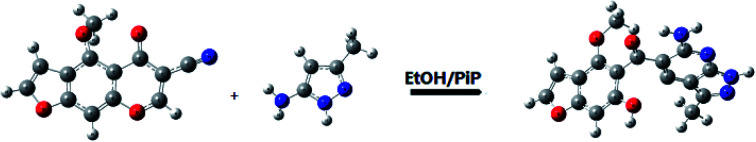

## 1. Introduction

The naturally occurring furochromones, also known as furanochromones, khellin, and visnagin, are extracted from the fruits and seeds of *Ammi visnaga* L.^1^ and used for the treatment of psoriasis, vitiligo, angina, kidney stones, and as a spasmolytic agent.^2–4^ They are extensively used as analgesic, anti-inflammatory,^5,6^ anticancer,^7,8^ anticonvulsant,^9^ antitubercular,^10^ and antimicrobial agents.^11^ Optimized geometries of some furo[3,2-*g*]chromenes have been investigated by DFT-theoretical calculations.^12,13^ Photodiode, photovoltaic, photoelectrical, photosensitivity electronic spectra, molecular docking, computational, and solvatochromic studies were carried on a range of furo[3,2-*g*]chromenes.^14–19^ Furthermore, biological properties,^20–23^ chemical reactivity,^24^ and material applications^25^ have all drawn attention to the chemistry of pyrazole and its derivatives. Because of its importance in providing the key functions of frequency shifting, optical modulation, optical switching, optical logic, and optic memory for the emerging technologies in areas such as telecommunications, signal processing, and optical interconnections, the non-linear optical (NLO) effect is at the forefront of current research.^26^ Molecular electrostatic potential (MESP) mapped onto the electron density surface concurrently presents molecular size, shape, and electrostatic potential in terms of color grading and represents a very suitable tool in the analysis of the molecular structure and physiochemical property relationship of molecules such as biomolecules and medicines.^27^ Different photophysical, photovoltaic, and optoelectronic properties of some organic molecules were determined using the CAM-B3LYP/6-31G (d,p) level of DFT.^28^ Modifications with end caps and π-linkers led to an improvement in the optoelectronic properties of the designed molecules to be used as acceptors for high-efficiency in organic solar cells.^29^

The current work aims to synthesize the novel 5-(6-hydroxy-4-methoxy-1-benzofuran-5-ylcarbonyl)-6-amino-3-methyl-1*H*-pyrazolo[3,4-*b*]pyridine (3, HMBPP) with the goal of providing a comprehensive explanation of molecular geometry, molecular vibration, and electronic characteristics. The density functional theory (DFT) method with the basis set 6-311++G(d,p) was used to analyze the natural bond orbital (NBO), molecular electrostatic potential (MESP), electronic absorption spectra, Mulliken atomic charges, global reactivity descriptors, and thermodynamic properties. The non-linear optical (NLO) properties of the current compound have also been studied due to growing interest in organic materials for nonlinear optical devices, revealing that the molecule is important in pharmaceutical chemistry as well as an attractive object for future studies of nonlinear optical properties. The presented structure is classified as an organic semiconductor of small molecule and specified as a π-conjugated nanostructure and has delocalization of electrons as well as a large extinction coefficient and good light gain.

## 2. Experimental

### 2.1. 5-(6-Hydroxy-4-methoxy-1-benzofuran-5-ylcarbonyl)-6-amino-3-methyl-1*H*-pyrazolo[3,4-*b*] pyridine (3, HMBPP)

A mixture of 4-methoxy-5-oxo-5*H*-furo[3,2-*g*] chromene-6-carbonitrile (1) (0.48 g, 2 mmol) and 5-amino-3-methyl-1*H*-pyrazole (2) (0.20 g, 2 mmol) in absolute ethanol (20 mL) containing piperidine (0.1 mL) was heated under reflux for 45 min. The yellow crystals so formed during heating were filtered and recrystallized from methanol to give compound (3, HMBPP), yield (0.52 g, 78%), m.p. 299–300 °C. IR (KBr, cm^−1^): 3425 (OH), 3355, 3314 (NH_2_), 3125 (NH), 3055 (CH_arom_), 2960, 2942, 2865 (CH_aliph_), 1657 (C=O), 1547 (C=C). ^1^H-NMR (300 MHz, DMSO-*d*_6_, *δ*): 2.37 (s, 3H, CH_3_), 3.88 (s, 3H, OCH_3_), 6.94 (s, 1H, H-7_benzofuran_), 7.18 (d, 1H, *J* = 1.8 Hz, H-3_furan_), 7.94 (d, 1H, *J* = 2.4 Hz, H-2_furan_), 8.80 (s, 1H, H-4_pyridine_), 9.41 (bs, 2H, NH_2_ exchangeable with D_2_O), 10.23 (bs, 1H, NH exchangeable with D_2_O), 10.81 (bs, 1H, OH exchangeable with D_2_O). ^13^C-NMR (100 MHz, DMSO-*d*_6_, *δ*): 15.9 (CH_3_), 58.0 (OCH_3_), 91.9 (C′-7), 105.6 (C′-3), 108.1 (C′-3a), 110.3 (C′-5), 113.7 (C′-3a), 116.5 (C-5), 136.9 (C-4), 139.9 (C-3), 145.8 (C′-2), 150.3 (C-7a), 153.8 (C-4), 157.6 (C-6) 160.1 (C′-6), 162.9 (C′-7a), 196.2 (C=O). Mass spectrum, *m*/*z* (*I*_r_%): 338 (M^+^, 70), 308 (42), 286 (24), 272 (22), 258 (38), 191 (100), 163 (62), 147 (44), 134 (47), 117 (33), 106 (30), 101 (17), 91 (73), 78 (34), 64 (23). Anal. calcd for C_17_H_14_N_4_O_4_ (338.32): C, 60.35; H, 4.17; N, 16.56%. Found: C, 60.15; H, 4.02; N, 16.20%.

### 2.2. Apparatus

A digital Stuart SMP3 apparatus was used for melting point determination. FTIR Nicolet IS10 spectrophotometer (cm^−1^) was applied for measuring the infrared spectra using KBr disks. Mercury-300BB apparatus was used for measuring the ^1^H NMR (300 MHz) and ^13^C NMR (100 MHz) spectra using DMSO-*d*_6_ as a solvent and TMS (*δ*) as the internal standard. GC-2010 Shimadzu gas chromatography instrument mass spectrometer (70 eV) was used for measuring the mass spectra. Elemental microanalyses were performed using PerkinElmer 2400II at the Chemical War Department, Ministry of Defense, Egypt. The purity of the synthesized compounds was checked by thin layer chromatography and elemental microanalysis. PerkinElmer Lambda 4B spectrophotometer with 1.0 cm fused quartz cells was used to record the electronic absorption spectra of the solutions in the range of 200–900 nm. Spectral analysis of transmittance and reflectance are performed in the wavelength range of 200–750 nm.

### 2.3. Solvents

Methanol as polar solvent and cyclohexane as non-polar solvent were utilized without purification in Merck, AR-grade.

### 2.4. Computational details

The Gaussian 09 program^30^ was used to do the computations in this work, and the results were evaluated using the Gauss-view 05 molecular visualization program.^31^ DFT^32–35^ using a hybrid functional B3LYP,^36^ combining the Lee–Yang–Parr correlation functional (LYP)^37^ with Beck's three parameter (local, non-local, Hartree–Fock) hybrid exchange functional (B3), and the Coulomb Attenuating Method (CAM-B3LYP)^37^ was used to obtain the optimized geometrical parameters, vibrational frequencies, UV-Vis spectra, electronic transitions, and electronic characteristics such as HOMO–LUMO energies for the title compound 3 (HMBPP). For a better representation of the polar bonding in molecules, the basis set 6-311++G(d,p) with ‘d’ polarization functions on heavy atoms and ‘p’ polarization functions on hydrogen atoms were utilized.^38^ Using the same level of theory, NMR chemical shifts were estimated using the gauge, including the atomic orbital (GIAO) approach.^39^ In the NBO basis, the donor–acceptor interactions were evaluated using the second order Fock matrix.^40^

## 3. Results and discussion

### 3.1. Chemistry

**Scheme 1:**
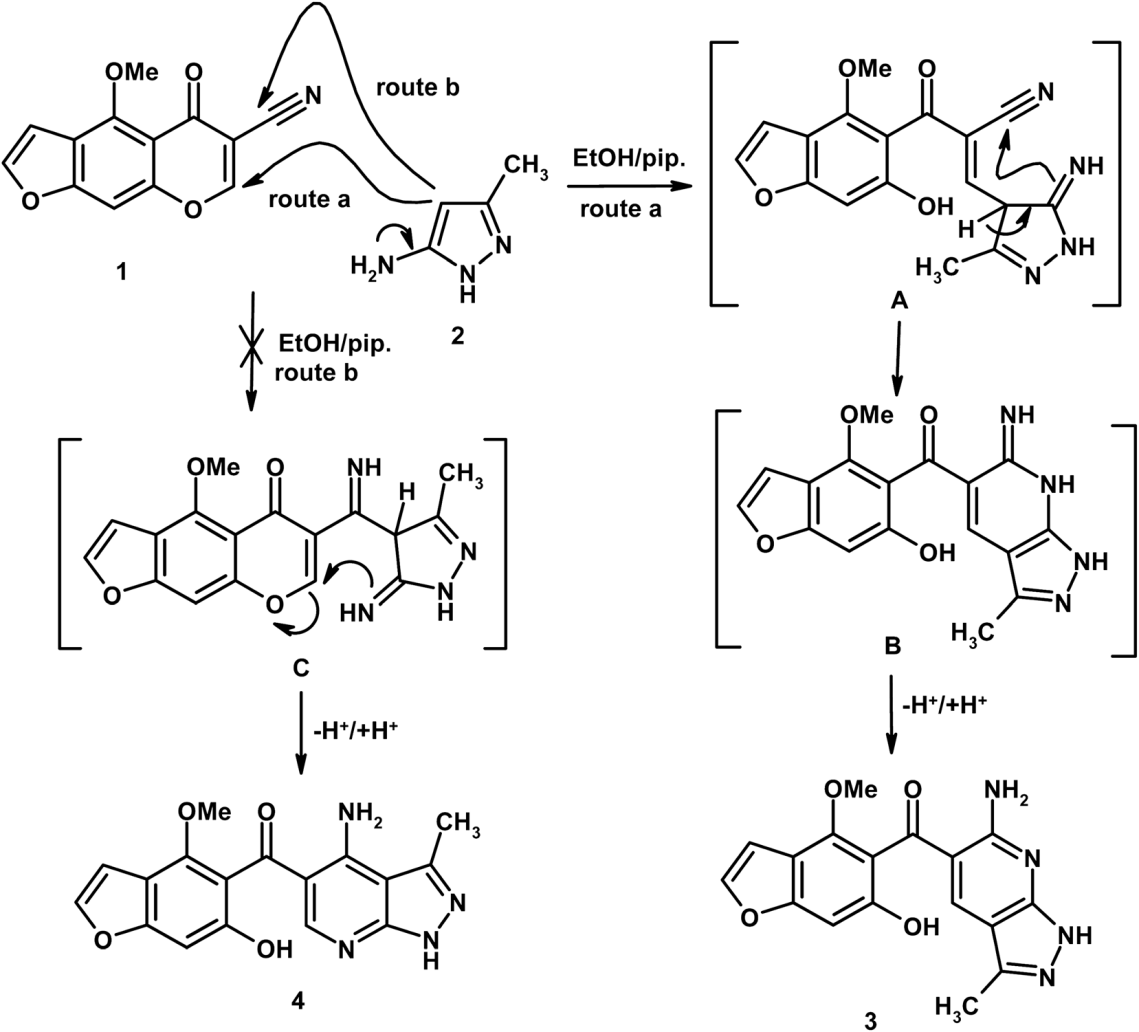
Formation of pyrazolo[3,4-*b*]pyridine derivative (3, HMBPP).

Reaction of 4-methoxy-5-oxo-5*H*-furo[3,2-*g*]chromene-6-carbonitrile (1) with 5-amino-3-methyl-1*H*-pyrazole (2) in boiling ethanol containing a few drops of piperidine afforded the novel 5-(6-hydroxy-4-methoxy-1-benzofuran-5-ylcarbonyl)-6-amino-3-methyl-1*H*-pyrazolo[3,4-*b*]pyridine (3), and another expected product 4 was ruled out (Scheme 1). Two reaction pathways for compounds 3 and 4 are depicted in Scheme 1. The nucleophilic reagent 2 usually begins to attack the electron deficient centers in compound 1, which is the C-7 position. Route a describes a nucleophilic attack at C-7 position with γ-pyrone ring opening giving intermediate A, which undergoes cycloaddition into the nitrile function giving intermediate B, followed by proton transfer giving product 3 (Scheme 1). If the reaction proceeds through route b, the nucleophilic reagent undergoes nucleophilic addition into the cyano group giving intermediate C, followed by nucleophilic attack at C-7 with ring opening and concomitant proton transfer giving compound 4. Due to the electron withdrawing action by mesomeric effect (at C-7) achieved by carbonyl and cyano groups and inductive effects achieved by atomic oxygen, the reaction prefers route a in which the nucleophilic attacks at the more electron deficient center (C-7 position) and route b will be excluded.

**Fig. 1:**
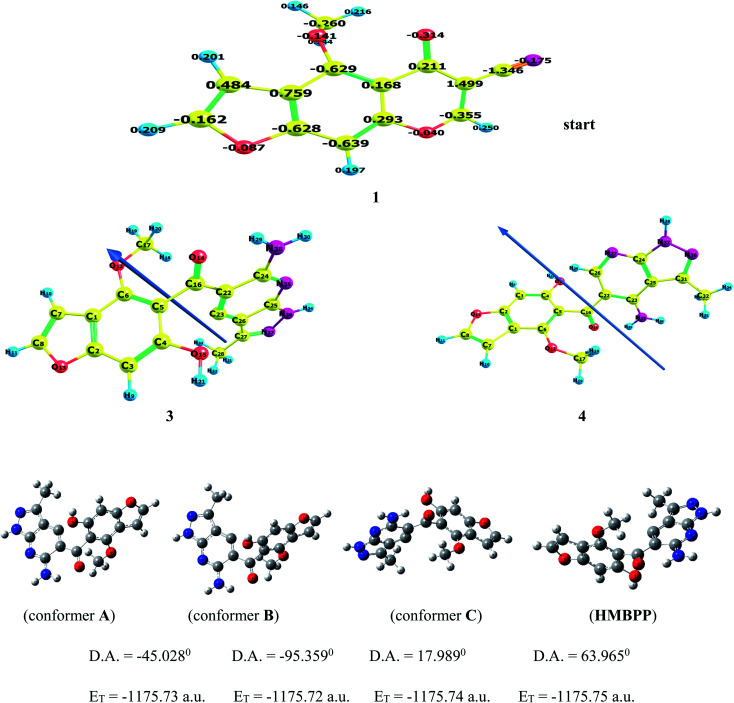
The optimized structure of compounds 1, 3 (HMBPP), and 4. Different conformers (A–C) by dihedral angle within the HMBPP structure using Gaussian program.

The above results were further confirmed by theoretical calculations, which calculate the charge density at C-7 and the cyano carbon, which show that the electron density charge on C-7 is higher than the carbon of the cyano group (*cf.*Fig. 1), so the reaction occurs through route a, giving product 3 and not the other expected compound 4 (Scheme 1). In addition, the computed results obtained show that compound 3 is more stable and highly reactive than compound 4 by (0.2317 eV, 5.3407 kcal mol^−1^) values. Also, compound 3 shows less hardness and more softness with high electrophilicity than compound 4 (*cf.*Table 1), so compound 3 is more stable and reactive than compound 4.

**Table:** Calculated *E*_LUMO_, *E*_HOMO_, energy band gap *E*_LUMO_ – *E*_HOMO_, ionization potential (IP), electron affinity (EA), electronegativity (*χ*), global hardness (*η*), chemical potential (*v*), global electrophilicity index (*ω*), global softness (*S*), and additional electronic charge (Δ*N*_max_) in eV for reactants 1 & 2 and expected products 3 & 4, using B3LYP/6-311++G(d,p)

Parameters	1	2	3, HMBPP	4	Ref. 13	Ref. 14	Ref. 15	Ref. 16
Energy of highest occupied molecular orbital (*E*_HOMO_)	−6.6248	−5.9734	−5.8077	−5.7544	−6.8416	−6.1431	−5.5262	−5.7865
Energy of lowest unoccupied molecular orbital (*E*_LUMO_)	−2.5832	−0.5809	−2.0756	−1.7906	−2.7257	−2.3574	−2.0231	−1.8006
Energy gap, (*E*_g_)	4.0416	5.3924	3.7321	3.9638	4.1159	3.7857	3.5030	3.9859
Dipole moment, (*μ*)	6.6237	3.1776	0.5390	1.9407	8.6600	7.9700	1.9141	5.3085
*I* (eV)	6.6248	5.9734	5.8077	5.7544	6.8416	6.1431	5.5262	5.7865
*A* (eV)	2.5832	0.5809	2.0756	1.7906	2.7257	2.3574	2.0231	1.8006
*χ* (eV)	4.6040	3.2772	3.9416	3.7725	4.7836	4.2502	3.7746	3.7936
*v* (eV^−1^)	−4.6040	−3.2772	−3.9416	−3.7725	−4.7836	−4.2502	−3.7746	−3.7936
*η* (eV)	2.0208	2.6962	1.8660	1.9819	2.0579	1.8928	1.7516	1.9929
*S* (eV^−1^)	0.2474	0.1854	0.2679	0.2523	0.2430	0.2641	0.2855	0.2509
*ω* (eV)	5.2446	1.9917	4.1629	3.5904	5.5596	4.7717	4.0674	3.6105
Δ*N*_max_	2.2783	1.2155	2.1123	1.9035	2.3245	2.2454	2.1549	1.9036

**Fig. 2:**
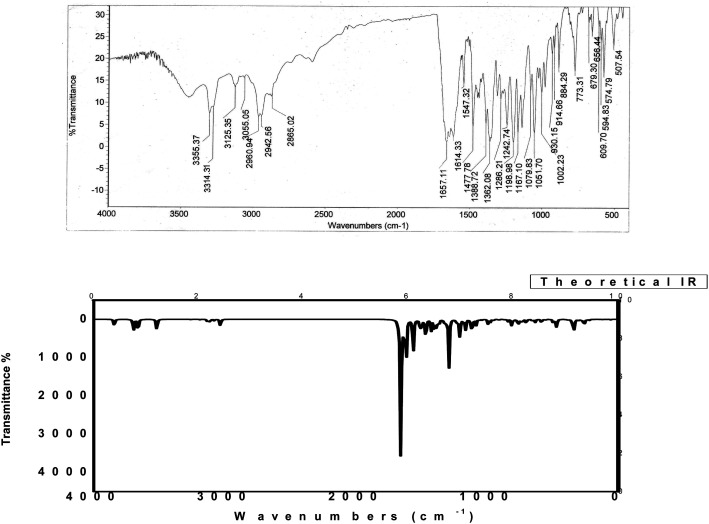
Experimental and calculated IR spectra of compound 3 (HMBPP) at B3LYP/6-311++G(d,p).

**Fig. 3:**
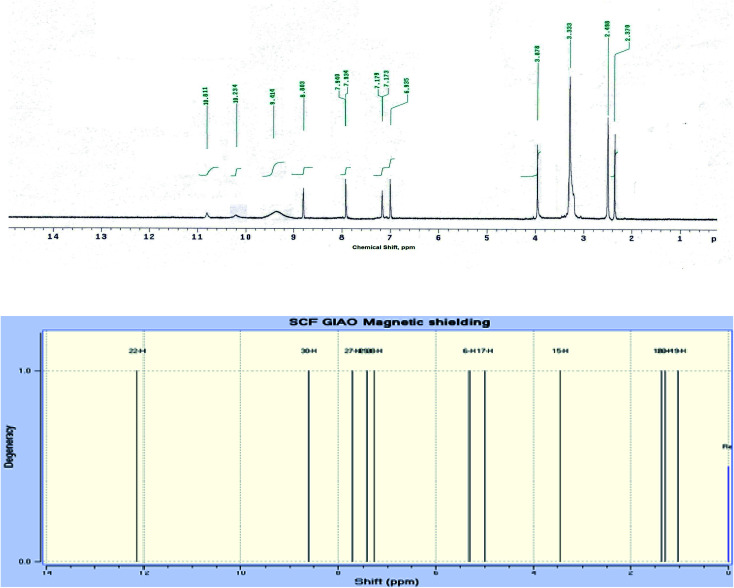
Experimental and calculated ^1^H NMR spectrum of compound 3 (HMBPP) at B3LYP/6-311++G(d,p).

**Fig. 4:**
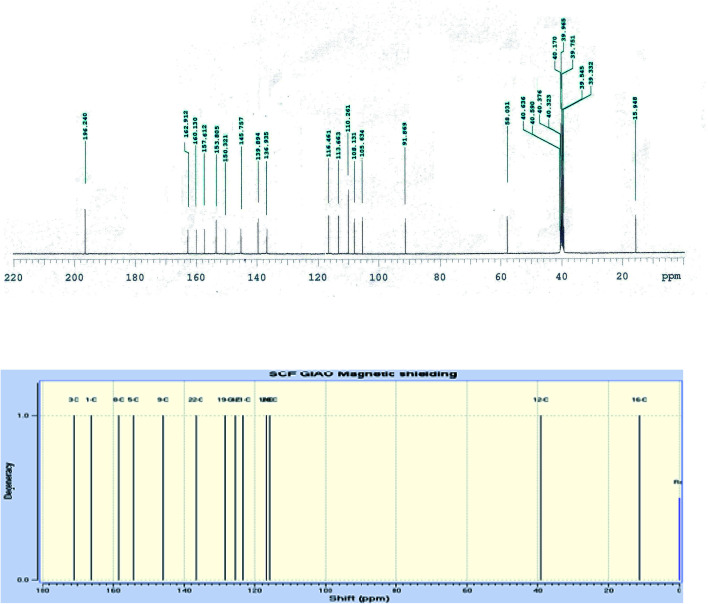
Experimental and calculated ^13^C NMR spectrum of compound 3 (HMBPP) at B3LYP/6-311++G(d,p).

**Fig. 5:**
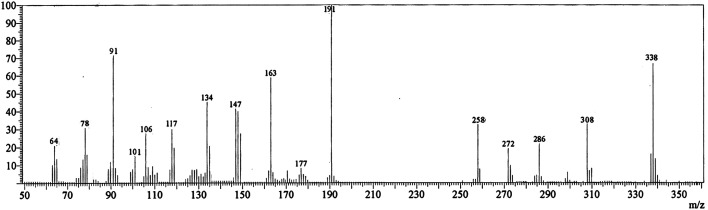
The mass spectrum of compound 3 (HMBPP).

**Scheme 2:**
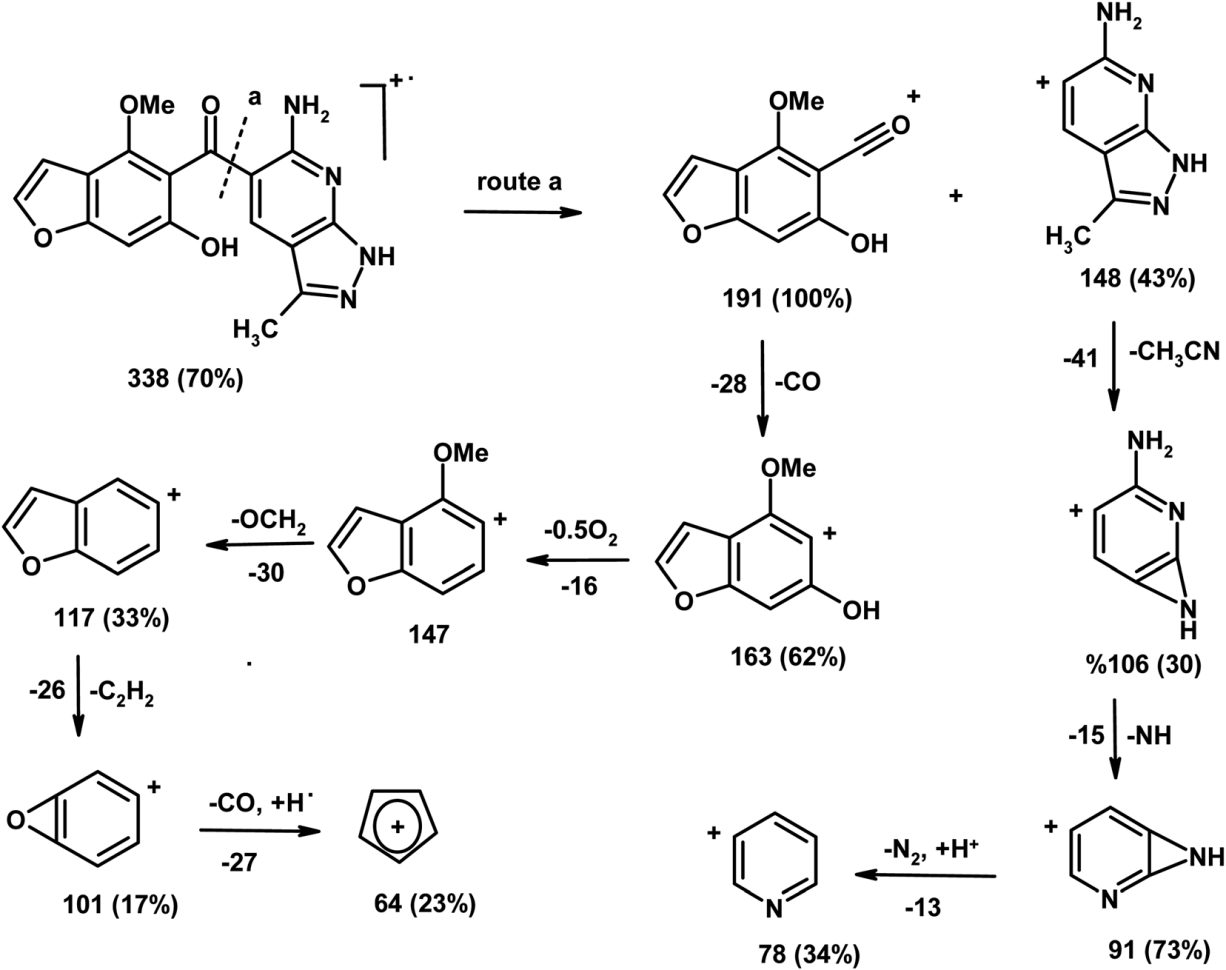
The mass fragmentation patterns of compound (3, HMBPP).

The IR spectrum of compound 3 (HMBPP) (Fig. 2) showed typical absorption bands at 3425 (OH), 3355, 3314 (NH_2_), 3125 (NH), 3055 (CH_arom_), 2960, 2942, 2865 (CH_aliph_), 1657 (C=O), and 1547 cm^−1^ (C=C). The ^1^H-NMR spectrum of compound 3 (HMBPP) (Fig. 3) revealed two characteristic doublets (*J* = 2.4 Hz) assignable to H-2_furan_ and H-3_furan_ at *δ* 7.94 and 7.18 ppm, respectively. The spectrum also revealed four singlet signals at *δ* 2.37 (CH_3_), 3.88 (OCH_3_), 6.94 (H-7_benzofuran_), and 8.80 (H-4_pyridine_). D_2_O exchangeable signals were observed at *δ* 9.41 (NH_2_), 10.23 (NH), and 10.81 (OH). The ^13^C NMR spectrum of compound 3 (HMBPP) (Fig. 4) revealed seventeen signals corresponding to the number of carbon atoms existing in the synthesized structure. These signals were characterized as follows; 15.9 (CH_3_), 58.0 (OCH_3_), 91.9 (C′-7), 105.6 (C′-3), 108.1 (C′-3a), 110.3 (C′-5), 113.7 (C′-3a), 116.5 (C-5), 136.9 (C-4), 139.9 (C-3), 145.8 (C′-2), 150.3 (C-7a), 153.8 (C-4), 157.6 (C-6) 160.1 (C′-6), 162.9 (C′-7a), 196.2 (C=O). The mass spectrum of compound 3 (HMBPP) (Fig. 5) recorded its molecular ion peak at *m*/*z* 338 and supports the identity of the structure. The mass fragmentation patterns of compound 3 (HMBPP) are depicted in Scheme 2.

### 3.2. Chemical reactivity

#### 3.2.1 Global reactivity descriptors

Global reactivity descriptors are useful for comparison of the reactivity of compounds 3 and 4, which are the energies of frontier molecular orbital (*E*_LUMO_, *E*_HOMO_), band gap (*E*_LUMO_ − *E*_HOMO_), ionization potential (IP), electron affinity (EA), electronegativity (*χ*), global hardness (*η*), chemical potential (*v*), global electrophilicity index (*ω*), global softness (*S*) and additional electronic charge ((Δ*N*_max_) and were calculated using eqn (1)–(8) and listed in Table 1.^41,42^

1 IP = −*E*_HOMO_

2 EA = −*E*_LUMO_

3 *χ* = (*E*_LUMO_ + *E*_HOMO_)/2

4 *v* = −*χ* = −(*E*_LUMO_ + *E*_HOMO_)/2

5 *η* = (*E*_LUMO_ − *E*_HOMO_)/2

6 *S* = 1/2*η*

7 *ω* = *v*^2^/2*η*

8 Δ*N*_max_ = −*v*/*η*

According to Parr *et al.*,^43^ the electrophilicity index (*ω*) is a global reactivity index, which is a positive and definite quantity like chemical hardness and chemical potential. When the system acquires an additional electronic charge (*N*) from the environment, this new reactivity index calculates the energy stabilization. Because an electrophile is a chemical species capable of accepting electrons from the environment, the direction of the charge transfer is completely determined by the electronic chemical potential of the molecule. As a result, when a molecule accepts an electronic charge, its energy must decrease, and its electronic chemical potential must be negative. The difference between the (Δ*N*_max_) values of interacting molecules is characterized as electrophilic charge transfer (ECT).^44^ If we consider two molecules 1 and 2 approaching each other (i) if ECT > 0, charge flows from 2 to 1 (ii) if ECT < 0, charge flows from 1 to 2. ECT is calculated using the equations given below:

9 ECT = (Δ*N*_max_)_1_ − (Δ*N*_max_)_2_

where

10 (Δ*N*_max_)_1_ = −*v*_1_/*η*_1_ and (Δ*N*_max_)_2_ = −*v*_2_/*η*_2_

Ionization potential (IP), electron affinity (EA), electronegativity (*χ*), global hardness (*η*), chemical potential (*v*), global electrophilicity index (*ω*), global softness (*S*), and additional electronic charge (Δ*N*_max_) were calculated for reactants 1 and 2 as well as for product 3 and another expected product 4, using the energies of frontier molecular orbitals (*E*_LUMO_, *E*_HOMO_), which are tabulated in Table 1. Electrophilic charge transfer (ECT) was calculated from the values of additional electron charge (Δ*N*_max_) of reactants 1 and 2 using eqn (9). The calculated value of ECT > 0 (ECT = 1.063) for reactant molecules indicates the flow of charge from nucleophile 2 into the electron deficient substrate 1. As a result, the reactant molecule 2 acts as a global nucleophile (electron donor) and substrate 1 as a global electrophile (electron acceptor). The nucleophilic behavior of compound 2 is favored by its low electrophilicity index and high chemical potential, whereas the electrophilic behavior of compound 1 is favored by its low chemical potential and high electrophilicity index. The higher electrophilicity index (*ω* = 4.163 eV) for product 3 than that of reactant 2 shows that it is a strong electrophile than reactant 2 and another expected product 4. So, product 3 is formed only through route (a) and is highly stable than another expected product 4, which is not formed (route (b)). The chemical softness, which is directly related to the stability of the molecule, showed a higher value (0.2679 eV^−1^) for product 3 as compared with the other expected product 4 and reactants (1 & 2), indicating higher stability of the formed product 3 (*cf.*Table 1). A comparison of the obtained results for the studied factors with those published for similar structures is listed in Table 1. The values of the obtained parameters for HMBPP are close to the values obtained for similar structures, considering the computational error percent, which confirms the accuracy of the obtained results.^13–16^

### 3.3. Molecular geometry

Density functional theory calculations using B3LYP and CAM-B3LYP functional with 6-311++G(d,p) basis set were used to determine the most relevant structural parameters (bond lengths, bond angles, and dihedral angles) of the title compound 3 (HMBPP) and are given in Table 2. Geometrical optimization was carried out without any symmetry constraints. The numbering of atoms in the molecules utilized in this paper is described in Fig. 1. Because experimental data is lacking, some of the most important structural parameters are compared to analogous systems for which crystal structures have been solved. The optimized structure of the title compound was compared to the experimental structure of a closely comparable molecule found in the literature.^45,46^ The agreement between the optimized and experimental crystal structure is quite good, indicating that the geometry optimization nearly replicates the observed conformation. The symmetry of the molecular structure of the title compound is C1. When compared to the ref. 45 and 46, the bond lengths and bond angles of the title molecule showed slight differences. The C–O bond length of the title compound is elongated, according to the calculations. As a result, it may be used to calculate a wide range of molecular and spectroscopic parameters, such as electronic properties, electric moments, and vibrational wavenumbers.

Selected optimized geometrical structure parameters for the studied compound 3 (HMBPP) computed at B3LYP/6-311++G(d,p) and CAM/B3LYP/6-311++G(d,p)

**Table:** 

Bond lengths (Å)	B3LYP/6-311++G(d,p)	CAM/B3LYP/6-311++G(d,p)	Exp. X-ray^45,46^	Bond angles (°)	B3LYP/6-311++G(d,p)	CAM/B3LYP/6-311++G(d,p)	Exp. X-ray^45,46^
C1–C6	1.352	1.375	1.374	∠C2O13C8	106.28	110.22	108.89
C1=C2	1.393	1.412	1.409	∠C6O12C17	123.26	122.52	124.24
C8–O13	1.372	1.384	1.355	∠C22C16O14	115.61	120.12	118.98
C17–O12	1.428	1.435	1.355	∠C1C7C8	105.87	108.56	112.54
C16=O14	1.229	1.241	1.248	∠C1C6C5	118.52	120.21	121.91
C24–N35	1.344	1.358	1.363	∠C24N35C25	116.22	117.45	118.23
N36–N37	1.374	1.382	1.431	∠N35C25N36	112.95	127.46	120.64
N20–H23	1.006	1.007	1.017	∠C27N36N37	106.81	108.94	109.57
O15–H21	0.962	0.975	0.989	∠C25N36H34	119.06	127.48	126.39
C7–H10	1.077	1.083	0.98	∠C8O13H11	106.08	115.11	111.96

**Table:** 

Dihedral angles (°)	B3LYP/6-311++G(d,p)	CAM/B3LYP/6-311++G(d,p)
∠O14C16C5C6	67.927	89.765
∠O14C16C22C24	5.710	7.852
∠C22C24N38H30	177.58	179.32
∠N38C24N35C25	−179.14	−179.87
∠N35C25N36H34	0.221	0.523
∠H10C7C8H11	−0.025	−0.342
∠C6C1C7C8	179.73	179.87

The stability of the current compound HMBPP was achieved from three different conformers (A–C) by variation of the dihedral angle within the molecule using the Gaussian program, as shown in Fig. 1. It was observed that the more stable conformer is C by a change in D.A = 17.989° and total energy value *E*_T_ = −1175.74 a.u. Compound HMBPP is more stable than conformer C by a difference in energy of 0.01 a.u.; (0.272 eV); (6.2696 kcal). It is also more stable than conformer B by 0.03 a.u.; (0.816 eV); (18.8088 kcal) and conformer A by 0.02 a.u.; (0.544 eV); (12.5392 kcal).

### 3.4. ^1^H NMR and ^13^C NMR spectroscopy

The ^1^H NMR and ^13^C NMR chemical shifts were computed using the GIAO method with the B3LYP and CAM-B3LYP functional and 6-311++G(d,p) basis set.^47^Table 3 and Fig. 3, 4 show the experimental and computed values of ^1^H NMR and ^13^C NMR chemical shifts of the title compound 3 (HMBPP). Except for the proton of the amino group, there is good agreement between the experimental and computed chemical shifts values in ^1^H NMR.^48^

**Table:** Experimental and calculated ^1^H and ^13^C NMR chemical shifts of compound 3 (HMBPP) using DFT/B3LYP and CAM-B3LYP/6-311++G(d,p)

Atom no.	^1^H-NMR calculated		Experimental	Atom no.	^13^C-NMR calculated		Experimental
	B3LYP/6-311++G(d,p)	CAM/B3LYP/6-311++G(d,p)			B3LYP/6-311++G(d,p)	CAM/B3LYP/6-311++G(d,p)	
H9	6.54	6.67	6.94	C1	106.62	106.82	105.6
H10	7.23	7.32	7.18	C2	107.58	107.87	108.1
H11	7.47	7.48	7.94	C3	112.64	112.75	113.7
H18	3.23	3.24	3.88	C4	136.65	138.55	136.9
H19	3.25	3.26		C5	109.56	109.66	110.3
H20	2.87	2.89		C6	115.62	115.75	116.5
H21	8.81	8.85	8.80	C7	88.97	88.99	91.9
H29	9.55	9.65	9.41	C8	140.85	140.89	139.9
H30	7.86	8.05	10.23	C16	190.32	194.65	196.2
H31	2.59	2.67	2.37	C17	66.05	66.06	58.0
H32	2.57	2.66		C22	143.62	144.76	145.8
H33	2.87	2.88		C23	148.25	149.35	150.3
H34	10.71	10.85	10.81	C24	150.32	152.81	153.8
				C25	155.35	156.78	157.6
				C26	158.72	159.86	160.1
				C27	160.52	161.92	162.9
				C28	23.05	24.88	15.9

From the computed and experimental chemical shift values, H18–H20 and H31–H33 have smaller values than the other protons H29, H30, and H34; this difference may be attributed to the electronic charge density around the ring. In the experimental ^13^C NMR spectrum (DMSO), the chemical shift values (*δ*) of carbon atoms are between 16–196 ppm. The molecule has seventeen carbons; however, these carbons are classified into three groups (attached with benzofuran, pyrazole, and pyridine), consistent with the structure and molecular symmetry.

### 3.5. UV-visible absorption spectroscopy

**Fig. 6:**
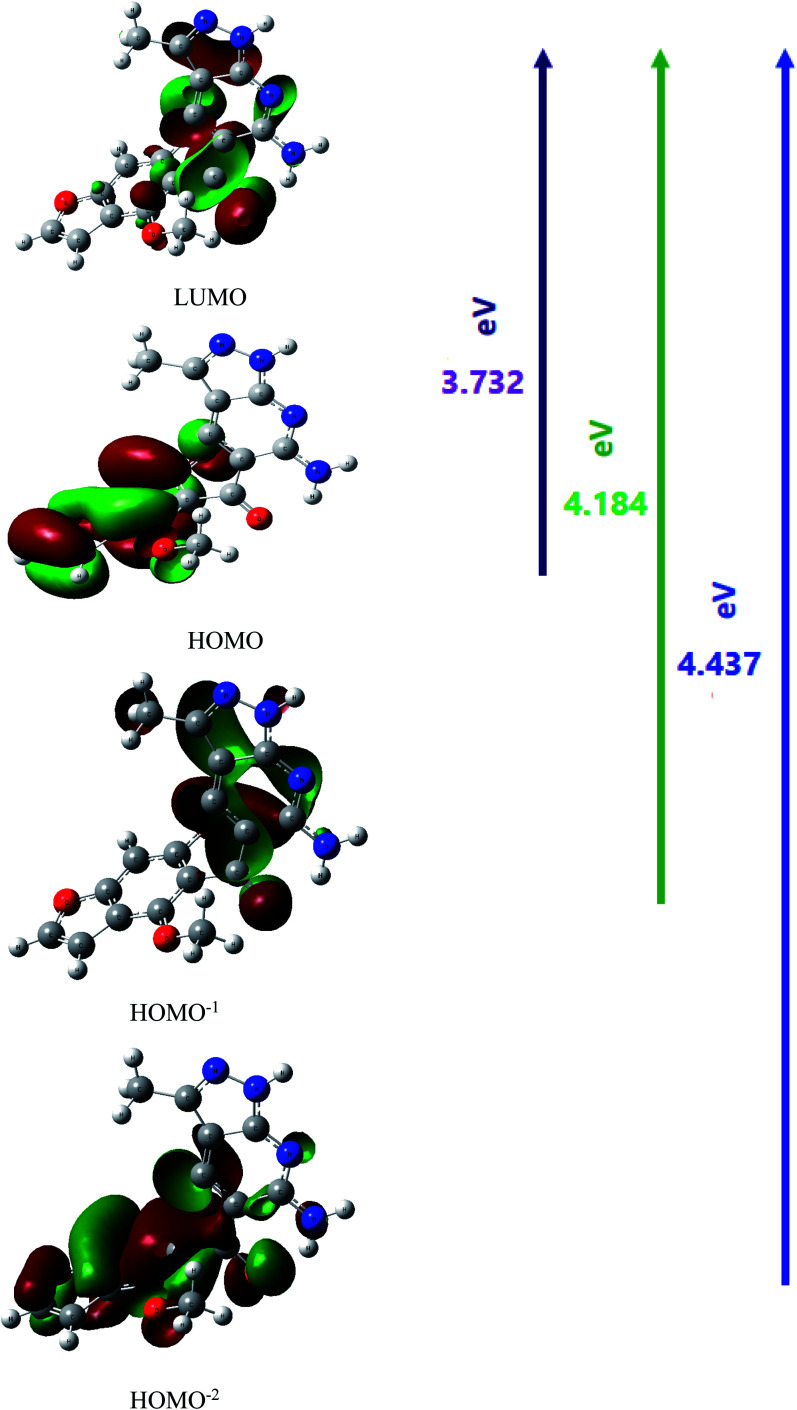
Molecular orbitals (HOMO → LUMO, HOMO^−1^ → LUMO and HOMO^−2^ → LUMO) of the compound 3 (HMBPP) at the B3LYP/6-311G(d,p) basis set.

**Fig. 7:**
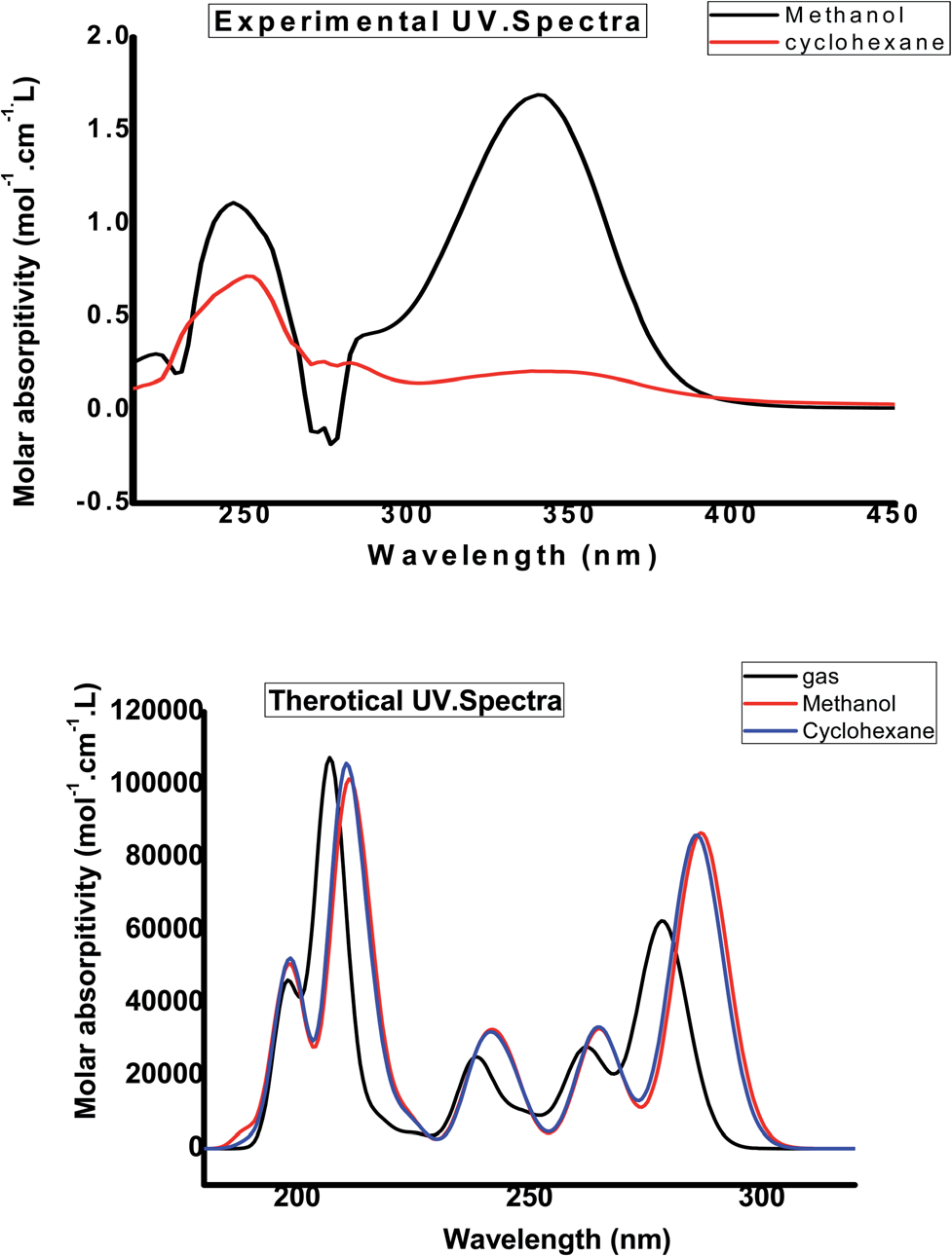
Experimental and calculated electronic absorption spectra of compound 3 (HMBPP) in different solvents.

The UV-Vis absorption spectrum was calculated theoretically using the TD-DFT method with the B3LYP/CAM-B3LYP functional and 6-311++G(d,p) basis set, with the solvent effect taken into account using the Integral Equation Formalism Polarizable Continuum Model (IEFPCM). Table 4 compares experimental UV data with the calculated UV data and the related properties, such as the vertical excitation energies, oscillator strength (*f*), percentage contribution of probable transition, and the corresponding absorption wavelength. The B3LYP functional predicts one intense electronic transition at 298 nm in cyclohexane with an oscillator strength *f* = 0.231, which agrees well with the measured experimental data (*λ*_max/nm_ = 355 nm in cyclohexane), as shown in Fig. 7. With a contribution of 49.6%, this electronic absorption corresponds to the transition from HOMO to LUMO. In the experimental UV spectrum of the studied molecule HMBPP, the ineffectual band around 265 nm in cyclohexane is an electronic transition from HOMO^−2^ to LUMO with 1.6% contribution and from HOMO^−1^ to LUMO with 47% contribution. In cyclohexane solvent, the corresponding theoretical peak in the TD-DFT UV spectrum is at 325 nm due to the n–π* transition. Fig. 6 describes the molecular orbitals and electronic transitions for HMBPP. The value of the energy gap between HOMO and LUMO is 3.732 eV. This shows the chemical reactivity of the compound HMBPP and proves the occurrence of eventual charge transfer within the molecule.

**Table:** Experimental and theoretical absorption wavelength *λ*_max/nm_, excitation energies *E* (eV) of compound 3 (HMBPP) using DFT/B3LYP and CAM-B3LYP/6-311++G(d,p)

States no.	Electronic transitions (molecular orbitals involved)	Energy (eV)	Calculated		Oscillatory strength (*f*)		Percentage contribution of probable transition		Observed *λ*_max/nm_
			B3LYP	CAM/B3LYP	B3LYP	CAM/B3LYP	B3LYP	CAM/B3LYP	
1	H → L	3.584	298	295	0.231	0.210	50.11	45.52	355
2	H^−2^ → L	4.585	260	250	0.151	0.163	2.71	3.66	265
	H^−1^ → L	5.548					48.1	39.75	

### 3.6. Vibrational assignment

The observed and computed wave numbers (scaled) with their assignments are depicted in Table 5. Due to the discard of anharmonicity present in the real system, the calculated vibrational wavenumbers are higher than the experimental values. So, calculated wavenumbers using B3LYP and CAM-B3LYP are scaled down by a single factor of 0.9679 and 0.9587,^49^ respectively, and compared with experimental wavenumbers. Fig. 2 shows the IR spectrum of compound HMBPP. At 3455 cm^−1^ (B3LYP) and 3423 cm^−1^ (CAM-B3LYP), the computed vibration is assigned to the asymmetric OH scissoring vibration for HMBPP, demonstrating similar agreement with the experimental findings at 3425 cm^−1^.

**Table:** Experimental and calculated vibrational frequencies in cm^−1^ of compound 3 (HMBPP) using DFT/B3LYP and CAM-B3LYP/6-311++G (d, p) with proposed assignments

No.	Exp.	Calculated		Vibrational assignments	References
		B3LYP	CAM/B3LYP		
1	3425	3455	3423	Scissoring O–H	49
2	3355, 3314	3368, 3339	3351, 3317	Asymmetric NH_2_ stretching	50
3	3125	3175	3144	Symmetric N–H stretching	50
4	3055	3078	3049	Asymmetric C–H_aromatic_ stretching	49
5	2960, 2942, 2865	2971, 2942, 2894	2943, 2914, 2867	Symmetric C–H_aliphatic_ stretching	48
6	1657	1704	1687	Symmetric C=O stretching	51
7	1547	1578	1563	C=C in plane bending	51

#### 3.6.1 Amino group (NH_2_) group vibrations

NH_2_ stretching vibrations of HMBPP are observed at 3355 and 3314 cm^−1^, respectively, which shows good agreement with the computed values, 3368, 3339 cm^−1^ for B3LYP level and 3351 and 3317 cm^−1^ for CAM-B3LYP level. The heteroaromatic molecule containing the NH_2_ group shows stretching absorption in the region 3500–3220 cm^−1^. The stretching modes for asymmetrical and symmetrical vibrations for the N–H appear near 3500-3400 cm^−1^.^50^ Herein, the N–H stretching of HMBPP is observed at 3125 cm^−1^ and computed values at 3175 and 3144 cm^−1^ for B3LYP and CAM-B3LYP levels, respectively.

#### 3.6.2 Carbonyl group (C=O) vibrations

The presence of a carbonyl group is indicated by strong bands in the FT-IR between 1690 and 1800 cm^−1^,^51^ which is caused by the C=O stretching motion. The bond strength, which is affected by the inductive, conjugative, field, and steric effects, determines the wavenumber of the C=O stretching vibration. The strong band at 1657 cm^−1^ in the FT-IR spectrum of the current molecule is assigned to the C=O stretching mode. The calculated C=O stretching mode by B3LYP level is 1704 cm^−1^ and by CAM-B3LYP level is 1687 cm^−1^, which agrees well with the experimental measurement.

#### 3.6.3 Aromatic C–H, aliphatic C–H, and C=C vibrations

The aromatic C–H stretching vibrations are normally found between 3100 and 3000 cm^−1^.^49^ The wavenumbers calculated at 3078 and 3049 cm^−1^ for B3LYP level and CAM-B3LYP level are assigned to the stretching vibration of C–H_aromatic_, which is observed experimentally at 3055 cm^−1^.

In aromatic hydrocarbons, skeletal vibrations involving C=C stretching within the ring are observed in the region between 1600 and 1585 cm^−1^.^51^ The wavenumbers calculated at 1578 cm^−1^ by B3LYP level and 1563 cm^−1^ by CAM-B3LYP level are assigned to the C=C stretching vibration in the benzene ring, which shows good agreement with the experimental value at 1547 cm^−1^.

Symmetric stretching vibrations of the CH_3_ group are expected in the range of 2900–3050 cm^−1^.^48^ The stretching mode of the methyl group (C–H_aliphatic_) is calculated to be at 2971, 2942, 2894 cm^−1^ by B3LYP level and 2943, 2914, 2867 cm^−1^ by CAM-B3LYP level, showing good agreement with the experimental values, 2960, 2942, and 2865 cm^−1^.

### 3.7. Molecular electrostatic potential

**Fig. 8:**
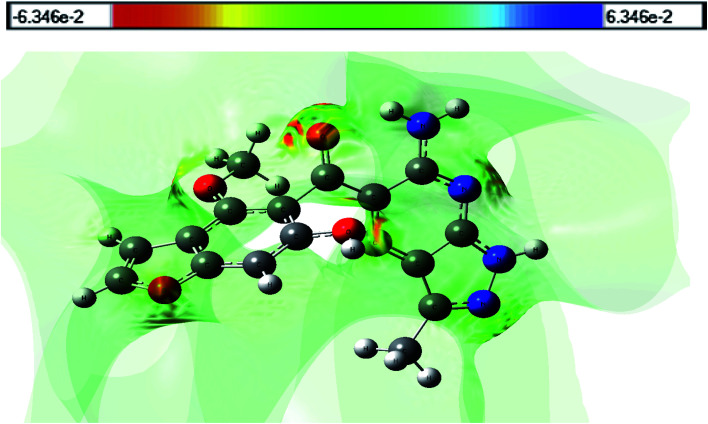
3D plots of the molecular electrostatic potential map of compound 3 (HMBPP).

Molecular electrostatic potential (MESP) can be utilized to estimate the electrophilic (electron rich region) and nucleophilic (electron poor region) reactive sites. The red and blue regions in the MESP denote electron rich and electron poor regions, respectively, while the green region denotes a nearly neutral region. Because the binding site, in general, is predicted to contain opposite areas of electrostatic potential, the variation in electrostatic potential produced by a molecule is largely responsible for the binding of medicine to its receptor binding sites. Fig. 8 shows an MESP map of the title compound (HMBPP) that was produced at the optimized geometry using Gauss view software. The most important negative potential region around the oxygen atom and nitrogen atom (nitrile group) is readily visible in the MESP of the molecule, which is characterized by yellowish red color, as is the binding site for electrophilic attack. Protons H21, H29, H30, and H34 have the highest positive potential charge, while the remainder of the molecule appears to have neutral electrostatic potential.

### 3.8. Natural bond orbital analysis

The calculations for the Natural Bond Orbital (NBO)^52^ were done with the Gaussian09 software and the B3LYP/6-311++G(d,p) technique. It provides a useful foundation for investigating charge transfer and conjugative interaction in molecular systems, as well as intramolecular and intermolecular bonding and bond interaction. The more intense the connection between electron donors and electron acceptors, *i.e.*, the more donating propensity from electron donors to electron acceptors and the greater the amount of conjugation of the overall system, the higher the stabilizing energy value. In the NBO study, the second order Fock matrix was used to analyze donor (*i*)–acceptor (*j*), *i.e.*, the interaction between donor and acceptor level bonds.^49^ The interaction results in a loss of occupancy from the idealized Lewis structure's electron NBO concentration to an empty non-Lewis orbital. The stabilization energy *E*^(2)^ associated with the delocalization *i* → *j* for each donor (*i*) and acceptor (*j*) is as follows:

11 *E*^(2)^ = Δ*E*_*j*_ = *q*_*i*_(*F*(*ij*)^2^/*ε*_*j*_ – *ε*_*i*_)

where *q*_*i*_ is the donor orbital occupancy, *ε*_*i*_ and *ε*_*j*_ are the diagonal elements, and *F*_*ij*_ is the off diagonal NBO Fock matrix element. The larger *E*^(2)^ value in NBO analysis, the concentrated the contact between electron-donors and electron-acceptors, and the higher the amount of conjugation of the entire system. Table 6 shows the possible intensive interaction in NBO. Strong intramolecular hyper conjugative interactions caused an increase in electron density (ED) and intramolecular charge transfer (ICT), leading to the stabilization of the system.

**Table:** Second order perturbation theory analysis of Fock matrix in NBO basis *E*^(2)^ values (kcal mol^−1^) for the optimized structure HMBPP

Donor	Type	ED(*i*)(e)	Acceptor	Type	ED(*i*)(e)	*E* ^(2)^ a (kcal mol^−1^)	*E*_(*j*)_ − *E*_(*i*)_^b^ (a.u)	*F*(*ij*)^c^ (a.u)
BD C1–C2	π	1.67064	BD*C3–C4	π*	0.44073	23.96	0.27	0.074
BD C1–C2	π	1.67064	BD*C5–C6	π*	0.34565	18.82	0.28	0.065
BD C3–C4	π	1.61301	BD*C8–C7	π*	0.36539	21.90	0.28	0.070
BD C5–C6	π	1.71294	BD*C1–C2	π*	0.28169	19.80	0.31	0.070
BD C8–C7	π	1.65791	BD*C23–C25	π*	0.23631	22.34	0.31	0.076
BDC23-C25	π	1.72899	BD* C8–C7	π*	0.36539	11.88	0.29	0.053
BD C4–C5	σ	1.96158	BD*C6–C26	σ*	0.03115	5.01	0.81	0.057
BDC16–C22	π	1.72899	BD*C5–C16	π*	0.41038	24.94	0.29	0.078
BDC5–C16	π	1.79357	BD*C27–N37	π*	0.10286	20.90	0.42	0.086
LP(2) O12		1.74650	BD*C3– C4	π*	0.44073	31.66	0.34	0.098
LP(2) O13		1.74650	BD*C17–O12	π*	0.26658	34.36	0.36	0.099
LP(2) O14		1.82201	BD*C22–C23	σ*	0.06794	17.92	0.69	0.102
LP(2) O15		1.82201	BD*C16–O14	σ*	0.12230	37.79	0.56	0.132
LP(1) N35		1.57902	BD* C8– C7	π*	0.36539	51.39	0.30	0.112
LP(1) N36		1.57902	BD*C5–C16	π*	0.41038	45.62	0.30	0.105
LP(1) N37		1.96981	RY*C28	σ*	0.01764	15.90	1.32	0.130
LP(1) N38		1.96915	BD*C26–C27	σ*	0.03481	12.59	1.00	0.100
BD*C3–C4	π*	0.44073	BD*C1–C2	π*	0.28169	154.07	0.02	0.083
BD*C5–C6	π*	0.34565	BD*C1–C2	π*	0.28169	183.38	0.01	0.080
BD*C8–C7	π*	0.36539	BD*C4–O15	π*	0.26658	144.50	0.01	0.074
BD*C16C22	π*	0.36539	BD*C23–C25	π*	0.23631	167.55	0.01	0.076
BD*C5–C16	π*	0.41038	BD*C23–C25	π*	0.23631	192.23	0.01	0.075

a *E* ^(2)^ means energy of hyper conjugative interactions (stabilization energy).

b Energy difference between donor and acceptor *i* and *j* NBO orbitals.

c *F*_(*i*,*j*)_ is the Fock matrix element between *i* and *j* NBO orbital. LP_(n)_ is a valence lone pair orbital (n) on atom.

• Between C_3_–C_4_ from N_36_ of n_1_ (N_36_) → π* (C_3_–C_4_), which increases ED (0.41e), leading to stabilization of 45.62 kcal mol^−1^.

• Between C_16_–O_14_ from N_37_ of n_1_ (N_37_) → π* (C_16_–O_14_), which increases ED (0.12e), leading to stabilization of 37.79 kcal mol^−1^.

• Between C_5_–C_6_ from O_15_ of n_2_ (O_15_) → π* (C_5_–C_6_), which increases ED (0.28e), leading to stabilization of 19.80 kcal mol^−1^.

• C_18_–C_19_ from O_13_ of n_2_ (O_13_) → π* (C_18_–C_19_), which increases ED (0.27e), leading to stabilization of 34.36 kcal mol^−1^.

• Between C_25_–C_26_ from N_38_ of n_1_ (N_38_) → σ* (C_25_–C_26_), which increases ED (0.36e), leading to stabilization of 51.39 kcal mol^−1^.

• Between C_26_–C_27_ from N_38_ of n_1_ (N_38_) → π* (C_26_–C_27_), which increases ED (0.035e), leading to stabilization of 12.59 kcal mol^−1^.

• Between C_19_–C_31_ from N_35_ of n_1_ (N_35_) → σ* (C_19_–C_31_), which increases ED (0.34e), leading to stabilization of 18.82 kcal mol^−1^.

• Between C_6_–C_7_ from O_12_ of n_2_ (O_12_) → σ* (C_6_–C_7_), which increases ED (0.44e), leading to stabilization of 31.66 kcal mol^−1^.

• Between C_7_–N_36_ from O_15_ of n_2_ (O_15_) → σ* (C_7_–N_36_), which increases ED (0.018e), leading to stabilization of 15.90 kcal mol^−1^.

The electron density is transferred from n(O), n(N) to antibonding π*, σ* orbital of C–N, C–C, C–O, explaining both the elongation and red shift.

### 3.9. Natural population analysis and natural charges

Table 7 shows the natural electronic configuration of HMBPP active sites at the B3LYP/6-311++G (d,p), together with the natural charge and population of total electrons on the subshells. O12, O13, O14, O15, N35, N36, N37, and N38 atoms are the most negative center atoms. Carbon atoms attached to these heteroatoms atoms are the most positive centers, as well as protons (H21, H29, H30, and H34), indicating a limited electron from the HMBPP molecule's static electricity. Moreover, HMBPP has 176 electrons that are coordinated in sub-shells as a total Lewis and a total non-Lewis in natural population analysis.

**Table:** Natural charge and natural population analysis for HMBPP

Atom no.	Natural charge	Natural population				Natural electronic configuration
		Core	Valence	Rydberg	Total	
O12	−0.55452	1.999	6.52654	0.02829	8.5545	[core]2S (1.59)2p (4.94)3p (0.02)
O13	−0.47311	1.999	6.45779	0.01560	8.4731	[core]2S (1.60)2p (4.86)3p (0.01)
O14	−0.68138	1.999	6.59382	0.08783	8.6814	[core]2S (1.70)2p (4.90)3S (0.02)
O15	−0.70428	1.999	6.67095	0.03358	8.7043	[core]2S (1.66)2p (5.02)3S (0.01)
N35	−0.62238	1.999	5.54292	0.08231	7.6224	[core]2S (1.35)2p (4.19)3p (0.06)
N36	−0.37010	1.999	5.34917	0.02163	7.3701	[core]2S (1.22)2p (4.13)3p (0.01)
N37	−0.30136	1.999	5.26316	0.03883	7.3014	[core]2S (1.43)2p (3.84)3S (0.01)
N38	−0.80316	1.999	5.74629	0.05754	7.8032	[core]2S (1.29)2p (4.45)3p(0.03)
H21	0.47041	0.000	0.52245	0.00714	0.5296	1S (0.52)
H29	0.42232	0.000	0.56798	0.00970	0.5777	1S (0.57)2S (0.01)
H30	0.39585	0.000	0.60122	0.00294	0.6041	1S (0.60)
H34	0.40392	0.000	0.58961	0.00647	0.5961	1S (0.59)
Core	49.97721 (99.9544% of 50)					
Valence Lewis	121.92939 (96.7694% of 126)					
Total Lewis	171.90660 (97.6742% of 176)					
Valence non-Lewis	3.78126 (2.306% of 176)					
Rydberg non-Lewis	0.30514 (0.186% of 176)					
Total non-Lewis	4.85718 (2.7598% of 176)					

### 3.10. Nonlinear optical analysis

The interaction of applied electromagnetic fields in various materials to generate new electromagnetic fields with altered wavenumber, phase, or other physical properties is known as nonlinear optics. Organic compounds that can efficiently manipulate photonic signals are crucial in technologies like optical communication, optical computing, and dynamic image processing.^52^ Based on the finite field technique, the first hyperpolarizability of the title compound was computed using the B3LYP and CAM-B3LYP/6-311++G(d,p) basis sets. We concentrated on the hyper-Rayleigh scattering (*β*_HRS_) and depolarization ratio (DR) among second order NLO characteristics^53^ and the complete equations for calculating the magnitude of total dipole moment *μ*_tot_, the average polarizability *α*_tot_, the first hyperpolarizability *β*_tot_, and the second hyperpolarizability *y*_tot_ using the *x*, *y*, *z* components are as follows:

12 *μ* = (*μ*_*x*_^2^ + *μ*_*y*_^2^ + *μ*_*z*_^2^)^1/2^

13 〈*α*〉 = 1/3(*α*_*xx*_ + *α*_*yy*_ + *α*_*zz*_)

14 Δ*α* = ((*α*_*xx*_ − *α*_*yy*_)^2^ + (*α*_*yy*_ − *α*_*zz*_)^2^ + (*α*_*zz*_ − *α*_*xx*_)^2^/2)^1/2^

15 〈*β*〉 = (*β*_*x*_^2^ + *β*_*y*_^2^ + *β*_*z*_^2^)^1/2^

where *β*_*x*_ = *β*_*xxx*_ + *β*_*xyy*_ + *β*_*xzz*_, *β*_*y*_ = *β*_*yyy*_ + *β*_*xxy*_ + *β*_*yzz*_, *β*_*z*_ = *β*_zzz_+ *β*_*xx*z_+ *β*_yyz_

16 〈*y*〉 = 1/5[*y*_*xxxx*_ + *y*_*yyyy*_ + *y*_*zzzz*_ + 2(*y*_*xxyy*_ + *y*_*xxzz*_ + *y*_*yyzz*_)]

17
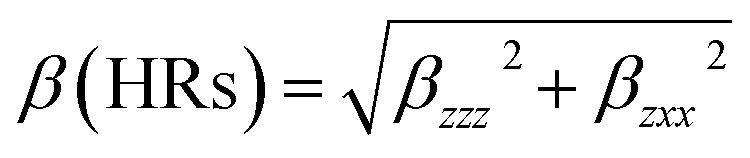
18
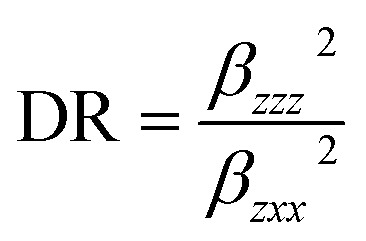


The calculated values have been converted into electrostatic units (esu) (*α*: 1 a.u. = 0.1482 × 10^−24^ esu; *β*: 1 a.u. = 8.6393 × 10^−33^ esu) because the value of the polarizabilities *α* and the hyperpolarizability of Gaussian output are reported in atomic mass units (a.u.). Table 8 shows the results of the electronic dipole moment *μ*_*i*_ (*i* = *x*, *y*, *z*), polarizability *α*_*ij*_, and first order hyperpolarizability *β*_*ijk*_. The computed dipole moment at the B3LYP level is 0.5390 D and 0.5533 D at the CAM-B3LYP level. *p*-Nitroaniline (PNA) is one of the prototypical molecules used in the study of the NLO properties of molecular systems. In this study, the typical NLO material, PNA was chosen as a reference molecule because there were no experimental values of the title compound in the literature. The calculated polarizability *α*_tot_, using B3LYP is 48.14 × 10^−24^ esu, while for CAM-B3LYP level is 45.89 × 10^−24^ esu, *i.e.*, two times greater than that of PNA molecule. The computed first hyperpolarizability *β*_tot_ of the current compound is 29.52 × 10^−33^ esu at B3LYP level and 11.82 × 10^−33^ esu for CAM-B3LYP level, which is higher (two times for B3LYP level and CAM-B3LYP level) than that of the common NLO material PNA (15.5 × 10^−33^ esu).^53–56^ In addition, the calculated second order hyperpolarizability *y* of HMBPP is −3.72 × 10^−35^ esu at B3LYP level and −3.06 × 10^−35^ esu at CAM-B3LYP level, *i.e.*, three times greater than that of PNA molecule. Furthermore, for the investigated compound, the lowest value of *β*, DR, and the highest value of *β*_HRS_ confirm the short bond length, indicating increased selectivity. We conclude that the title compound is an attractive object for future studies of nonlinear optical properties.

**Table:** Total static dipole moment (*μ*), mean polarizability (〈*α*〉), anisotropy of the polarizability (Δ*α*), mean first-order hyperpolarizability (〈*β*〉), and second order hyperpolarizability (〈*y*〉), for HMBPP using DFT/B3LYP and CAM-B3LYP/6-311++G(d, p)

	PNA	B3LYP	CAM-B3LYP	First-order hyperpolarizability (〈*β*〉)	PNA	B3LYP	CAM-B3LYP	Second-order hyperpolarizability (〈*y*〉)	PNA	B3LYP	CAM-B3LYP
**Dipole moment (*μ*)**											
*μ*_*x*_, D		−0.3398	−0.3398	*β*_*xxx*_, a.u.		16.3517	−4.1495	*y*_*xxxx*_, a.u.		−7225.6	−5309.5
*μ*_*y*_, D		0.3836	0.4036	*β*_*xxy*_, a.u.		11.6610	5.42152	*y*_*yyyy*_, a.u.		−2428.1	−2190.0
*μ*_*z*_, D		−0.1668	−0.1668	*β*_*xyy*_, a.u.		−8.1354	4.6657	*y*_*zzzz*_, a.u.		−996.85	−851.58
*μ*, Debye^a^	2.44 Debye^a^	0.5390	0.5533	*β*_*yyy*_, a.u.		3.6137	1.0785	*y*_*xxyy*_, a.u.		−1833.6	−1611.8

**Polarizability (〈*α*〉)**											
*α*_*xx*_, a.u.		54.5412	51.0592	*β*_*xxz*_, a.u.		−3.0052	2.4280	*y*_*xxzz*_, a.u.		−1532.1	−1420.7
*α*_*xy*_, a.u.		−2.4712	1.6904	*β*_*xyz*_, a.u.		0.6235	0.0934	*y*_*yyzz*_, a.u.		−602.31	−435.62
*α*_*yy*,_ a.u.		52.3629	50.5629	*β*_*yyz*_, a.u.		1.0981	0.5441	〈*y*〉 a.u.		−3717.3	−3057.5
*α*_*zz*_, a.u.		3.0547	2.5128	*β*_*xzz*_, a.u.		−0.1111	0.1534	〈*y*〉 × 10^−38^ esu	1.271 × 10^−35^ esu^d^	−3.7173 × 10^−35^ esu	−3.0575 × 10^−35^ esu
*α*_*yz*_, a.u.		4.2570	4.2539	*β*_*yzz*_, a.u.		1.4239	1.2407				
*α*_*xz*_, a.u.		37.5223	36.0492	*β*_*zzz*_, a.u.		0.2397	0.0709				
〈*α*〉 × 10^−24^ esu	22 × 10^−24^ esu^b^	48.1417	45.8904	〈*β*〉 × 10^−33^ esu	15.5 × 10^−30^ esu^c^	**29.5213**	**11.8237**				
Δ*α* ×10^−24^ esu		56.6541	55.2560	DR		0.4980	0.5250				
				*β*_HRS_		58.3240	60.3210				

a PNA results are taken from ref. 53.

b PNA results are taken from ref. 54.

c PNA results are taken from ref. 55.

d PNA results are taken from ref. 56.

### 3.11. Thermodynamic properties

**Fig. 9:**
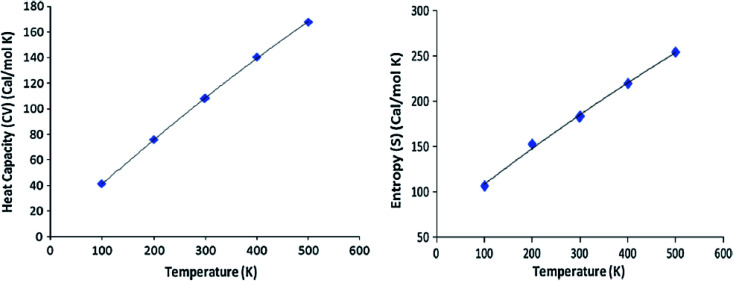
Correlation graphs of heat capacity and entropy calculated at various temperature using B3LYP/6-11G(d,p) of compound 3 (HMBPP).

**Fig. 10:**
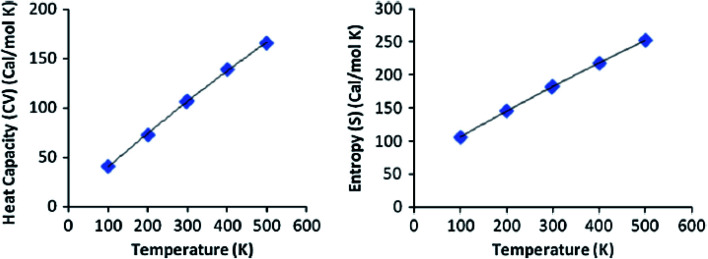
Correlation graphs of heat capacity and entropy calculated at various temperature using CAM/B3LYP/6-311G(d,p) of compound 3 (HMBPP).

At the HF and DFT levels using B3LYP/CAM-B3LYP functional with 6-311++G(d,p) basis set, the values of some thermodynamic parameters of the current compound, including zero-point vibrational energy, rotational temperatures, rotational constants, and energies at standard temperature 298 K were obtained (Table 9). Table 10 shows the standard statistical thermodynamic functions, heat capacity (CV), and entropy (*S*) for the title compound at various temperatures (100–500 K) using vibrational analysis at DFT/B3LYP and CAM-B3LYP methods with 6-311++G(d,p) basis set. When calculated in HF rather than B3LYP or CAM-B3LYP, the total energy, translational, rotational, and vibrational values are slightly higher. In all cases, the rotational constants and rotational temperature values are the same since the molecular vibrational intensities increase with temperature. Conventional statistical thermodynamic functions increase with temperatures ranging from 100 to 500 K.^57^ Quadratic formulas were used to fit the correlation equations between heat capacities, entropies, and temperatures, and the subsequent fitting factors (*R*^2^) for these thermodynamic parameters are given in eqn (19)–(22). The resultant fitting equations are as observed, and the correlation graphics are presented in Fig. 9 and 10.

19 CV = 4.3009 + 0.3776*T* − 1.10^−4^*T*^2^; (*R*^2^ = 0.9998) using B3LYP

20 *S* = 67.037 + 0.4242*T* – 0.0001*T*^2^; (*R*^2^ = 0.9974) using B3LYP

21 CV = 4.5000 + 0.3648*T* − 8.10^−5^*T*^2^; (*R*^2^ = 0.9996) using CAM/B3LYP

22 *S* = 66.086 + 0.4100*T* – 8.10^−5^*T*^2^; (*R*^2^ = 1) using CAM/B3LYP

**Table:** Calculated thermodynamic parameters of the title compound 3, HMBPP

Parameters	B3LYP/6-311++G(d,p)	CAM/B3LYP/6-311++G(d,p)	HF/6-311++G(d,p)
Zero-point vibrational energy (kcal mol^−1^)	175.73761	178.90704	191.24474
Rotational temperature (K)	0.01817	0.01828	0.01817
	0.00697	0.00699	0.00697
	0.00606	0.00608	0.00606
Rotational constant (GHZ)			
*X*	0.37856	0.38082	0.37856
*Y*	0.14537	0.14595	0.14537
*Z*	0.12625	0.12670	0.12606
Total energy *E*_total_ (kcal mol^−1^)	189.340	194.339	206.246
Translational	0.889	0.889	0.889
Rotational	0.889	0.889	0.889
Vibrational	187.563	192.561	204.469

**Table:** Thermodynamic functions at different temperatures at the B3LYP and CAM-B3LYP/6-311++G(d,p) level

Temperature (*T*) (K)	Heat capacity (CV) (cal mol^−1^ K^−1^)		Entropy (*S*) (cal mol^−1^ K^−1^)	
	B3LYP/6-311++G(d,p)	CAM-B3LYP/6-311++G(d,p)	B3LYP/6-311++G(d,p)	CAM-B3LYP/6-311++G(d,p)
100	42.20	45.807	108.49	107.421
200	76.86	75.901	155.76	146.630
298	108.617	107.512	185.90	183.652
300	109.25	108.652	183.65	185.352
400	141.47	140.457	220.87	219.318
500	168.83	167.863	257.65	254.527

These thermodynamic data could be useful for further research into the title compound. They can be used to calculate other thermodynamic energies using thermodynamic function relationships and to estimate chemical reaction directions using the second law of thermodynamics in the thermos chemical field.^58^ All thermodynamic calculations were performed in the gas phase and could not be applied to a solution.

### 3.12. Local reactivity descriptors

To model chemical reactivity and site selectivity, Fukui function (FF) is one of the extensively utilized local density functional descriptors. Fukui functions are determined using Hirshfeld population analysis of neutral, cation, and anion states of the molecule, using the following equations:

23 *f*_*k*_^+^ = [*q*_(*N*+1)_ – *q*_(*N*)_] for nucleophilic attack

24 *f*_*k*_^−^ = [*q*_(*N*)_ – *q*_(*N*−1)_] for electrophilic attack

25 *f*^0^_*k*_ = 1/2[*q*_(*N*+1)_ – *q*_(*N*−1)_] for radical attack

The total electrons present in the neutral, anion, and cation states of the molecule are (*N*, *N* − 1, *N* + 1), respectively. Additionally, electrophilicity indices (*ω*_*k*_^+^, *ω*_*k*_^−^, *ω*^0^_*k*_) and local softness (*s*_*k*_^+^, *s*_*k*_^−^, *s*^0^_*k*_) are used to define the reactivity of atoms in a molecule. The equivalent condensed to atom variations of the Fukui function is used to define these local reactivity descriptors associated with a site *k* in a molecule, using the following equations:

26 *s*_*k*_^+^ = *Sf*_*k*_^+^, *s*_*k*_^−^ = *Sf*_*k*_^−^, *s*^0^_*k*_ = *Sf*^0^_*k*_

27 *ω*_*k*_^+^ = *ωf*_*k*_^+^, *ω*_*k*_^−^ = *ωf*_*k*_^−^, *ω*^0^_*k*_ = *ωf*^0^_*k*_

Nucleophilic, electrophilic, and radical attacks are indicated by the +, −, and 0 signs, respectively. The highest values of all the three local reactivity descriptors (*f*_*k*_^−+^, *s*_*k*_^+−^, *ω*_*k*_^−+^) reveal that the site is more susceptible to nucleophilic or electrophilic attack than other atomic sites in reactants. Table 11 lists the Fukui functions (*f*_*k*_^+^, *f*_*k*_^−^), local softness (*s*_*k*_^+^, *s*_*k*_^−^) and local electrophilicity indices (*ω*_*k*_^+^, *ω*_*k*_^−^)^59^ for selected atomic sites of the molecule. In the product, the relatively high value of local reactivity descriptors (*f*_*k*_^+^, *s*_*k*_^+^, *ω*_*k*_^+^) at C4, C7, C17, C26, C27, and C28 in Table 11 indicate that these sites are prone to nucleophilic attack, whereas the relatively high value of local reactivity descriptors (*f*_*k*_^−^, *s*_*k*_^−^, *ω*_*k*_^−^) at N35, N36, N37, N38, and O15 indicates that this site is more prone to electrophilic attack. Thus, the produced molecule can be employed as an intermediate for the creation of new heterocyclic compounds.

**Table:** Using Hirshfeld population analysis: Fukui functions (*f*_*k*_^+^, *f*_*k*_^−^), local softness's (*s*_*k*_^+^, *s*_*k*_^−^) in eV, local electrophilicity indices (*ω*_*k*_^+^, *ω*_*k*_^−^) in eV for selected atomic sites of product

Atom no.	Hirshfield atomic charges			Fukui functions		Local softness's		local electrophilicity indices	
	*q*_*N*_	*q*_*N*+1_	*q*_*N*−1_	*f*_*k*_^+^	*f*_*k*_^−^	*s*_*k*_^+^	*s*_*k*_^−^	*ω*_*k*_^+^	*ω*_*k*_^−^
C1	−0.04103	0.040349	−0.09930	0.081374	0.058253	0.023598	0.016893	0.235985	0.168934
C2	0.060868	0.025310	−0.08050	−0.03556	0.141355	−0.01031	0.040993	−0.10312	0.409930
C3	0.045687	0.031721	0.041993	−0.01397	0.003694	−0.00405	0.001071	−0.04050	0.010713
C4	0.153426	0.326989	0.293155	0.173563	−0.13973	0.050333	−0.04052	0.503333	−0.40521
C5	0.265159	0.314418	0.229713	0.049259	0.035446	0.014285	0.010279	0.142851	0.102793
C6	0.027473	−0.00522	−0.03549	−0.03270	0.062961	−0.00948	0.018259	−0.09482	0.182587
C7	0.574829	0.596478	0.549941	0.021649	0.024888	0.006278	0.007218	0.062782	0.072175
C8	−0.54938	−0.70087	−0.71017	−0.15148	0.160783	−0.04393	0.046627	−0.43930	0.466271
C16	0.008188	0.031931	−0.06104	0.023743	0.069230	0.006885	0.020077	0.068855	0.200767
C17	0.089409	0.182182	0.166564	0.092773	−0.07716	0.026904	−0.02237	0.269042	−0.22375
C22	0.014894	0.011008	−0.05543	−0.00389	0.070322	−0.00113	0.020393	−0.01127	0.203934
C23	−0.01873	0.050243	−0.03541	0.068974	0.016674	0.020002	0.004835	0.200025	0.048355
C24	0.021178	0.024877	−0.09033	0.003699	0.111504	0.001073	0.032336	0.010727	0.323362
C25	0.041741	0.033533	−0.09006	−0.00821	0.131796	−0.00238	0.038221	−0.02380	0.382208
C26	0.173936	0.348567	0.332348	0.174631	−0.15841	0.050643	−0.04594	0.506430	−0.45939
C27	0.056988	0.292866	0.142892	0.235878	−0.08590	0.068405	−0.02491	0.684046	−0.24912
C28	0.036108	0.290717	0.142119	0.254609	−0.10601	0.073837	−0.03074	0.738366	−0.30743
O12	−0.55475	−0.54675	−0.55887	0.008009	0.004111	0.002323	0.001192	0.023226	0.011922
O13	−0.52328	−0.51435	−0.58219	0.008938	0.058910	0.002592	0.017084	0.025920	0.170839
O14	0.387000	0.554007	0.521021	0.167007	−0.13402	0.048432	−0.03887	0.484320	−0.38866
O15	0.322115	0.234416	0.200834	−0.08770	0.121281	−0.02543	0.035171	−0.25433	0.351715
N35	−0.38235	−0.43576	−0.50594	−0.05341	0.123585	−0.01549	0.035840	−0.15488	0.358397
N36	−0.03845	−0.02274	−0.13849	0.015705	0.100041	0.004554	0.029012	0.045545	0.290119
N37	−0.46628	−0.47695	−0.56499	−0.01067	0.098708	−0.00309	0.028625	−0.03095	0.286253
N38	0.188505	0.189793	0.223414	0.001288	−0.03491	0.000374	−0.01012	0.003735	−0.101240

## 4. Conclusion

A novel 5-(6-hydroxy-4-methoxy-1-benzofuran-5-ylcarbonyl)-6-amino-3-methyl-1*H*-pyrazolo[3,4-*b*] pyridine (3, HMBPP) was efficiently synthesized from the reaction of 6-formylvisnagin with 5-amino-3-methyl-1*H*-pyrazole (2). DFT theory was utilized to calculate the optimized geometric parameters (bond lengths, bond angles, and dihedral angles), which are compared to experimental data. Theoretical ^1^H and ^13^C chemical shift values (relative to TMS) are described and compared with experimental data, revealing excellent agreement for both ^1^H and ^13^C chemical shift values. The electronic properties are also computed and compared with the experimental UV-Vis spectra. The electron transition HOMO → LUMO (n → π*) determined the lowest singlet excited state of the molecule. The charge transfer within the molecule was represented in the NBO data. The calculated first hyperpolarizability of the title compound is 29.52 × 10^−33^ esu at the B3LYP level and 11.82 × 10^−33^ esu at the CAM-B3LYP level, which is higher (two times for B3LYP level and CAM-B3LYP level) than that of the common NLO material PNA (15.5 × 10^−33^ esu). In addition, the calculated second order hyperpolarizability 〈*y*〉 of HMBPP is −3.72 × 10^−35^ esu at the B3LYP level and −3.06 × 10^−35^ esu at the CAM-B3LYP level, *i.e.*, three times greater than that of PNA molecule, indicating the title molecule to be a potential candidate for nonlinear optical applications. In addition, the thermodynamic parameters and electronic absorption properties of the studied compound have been calculated. All theoretical results show good agreement with experimental data. The electronic absorption spectra computed theoretically using the Coulomb-attenuating method (CAM-B3LYP) in the gas phase and with the Corrected Linear Response Polarizable Continuum Model (CLRPCM) in cyclohexane and methanol indicate a good agreement with the observed spectra. Fukui functions, local softness, and electrophilicity indices for selected atomic sites have been calculated. The local reactivity descriptors (*f*_*k*_^−^, *s*_*k*_^−^, *ω*_*k*_^−^) at N35, N36, N37, N38, and O15 revealed these sites are more prone to electrophilic attack. Hence, the title molecule may be used as a precursor for the synthesis of new heterocyclic compounds having various biological activities.

## Conflicts of interest

There are no conflicts to declare.

## Supplementary Material

## References

[cit1] Abu-Hashem A. A., El-Shazly M. (2015). Synthesis, Reactions and Biological Activities of Furochromones: A Review. Eur. J. Med. Chem..

[cit2] Dewar H. A., Grimson T. A. (1950). Khellin in the treatment of angina of effort. Br. Heart J..

[cit3] Vedaldi D., Caflleri S., Dall'Acqua F., Andrea L., Bovalini L., Martelli P. (1988). Action mechanism of khellin on the stomach; khellin and hepato-renal lesions. Farmaco.

[cit4] Vanachayangkul P., Byer K., Khan S., Butterweck V. (2010). An aqueous extract of Ammi visnaga fruits and its constituents khellin and visnagin prevent cell damage caused by oxalate in renal epithelial cells. Phytomedicine.

[cit5] Ghate M., Kulkarni M. V. (2005). Synthesis and anti-inflammatory activity of 4-(5′-acetyl-6′-hydroxy-3′-methylbenzofuran-2′-yl) coumarin and 6-acetyl-3,7-dimethyl-2-(coumarin-4′yl)furo[3,2-g]chromen-5-one. Ind. J. Chem*.*.

[cit6] Frasinyuk M. S., Gorelov S. V., Bondarenko S. P., Khilya V. P. (2009). Synthesis and properties of 4-(3-amino-2-benzofuranyl) coumarins. Chem. Heterocycl. Comp..

[cit7] Ibrahim M. A., Al-Harbi S. A., Allehyani E. S., Alqurashi E. A., Alqarni A. O. (2022). Utility of 3-chloro-3-(4,9-dimethoxy-5-oxo-5H-furo[3,2-g]chromen-6-yl)prop-2-enal for construction of novel heterocyclic systems: synthesis, characterization, antimicrobial and anticancer evaluation. Synth. Commun..

[cit8] Amin K. M., Syam Y. M., Anwar M., Ali H. I., Abdel-Ghani T. M., Serry A. M. (2018). Synthesis, and molecular docking study of new benzofuran and furo[3,2-g] chromone-based cytotoxic agents against breast cancer and p38α MAP kinase inhibitors. Bioorg. Chem..

[cit9] Ragab F. A., El-Sayed N. A., Eissa A. A. M., El-Kerdawy A. M. (2010). Synthesis and anticonvulsant activity of certain substituted furochromone, benzofuran and flavone derivatives. Chem. Pharm. Bull*.*.

[cit10] Laxmi S. V., Reddy Y. T., Kuarm B. S., Reddy P. N., Crooks P. A., Rajitha B. (2011). Synthesis and evaluation of chromenyl barbiturates and thiobarbiturates as potential antitubercular agents. Bioorg. Med. Chem. Lett..

[cit11] Ibrahim M. A., Al-Harbi S. A., Allehyani E. (2020). Synthesis and Antimicrobial Evaluation of the Novel Heteroannulated Furo[3′,2′:6,7]chromeno[2,3-b]pyridines: Part 1. J. Heterocycl. Chem..

[cit12] Akchurin I. O., Yakhutina A. I., Bochkov A. Y., Solovjova N. P., Medvedev M. G., Traven V. F. (2018). Novel push-pull fluorescent dyes–7-(diethylamino) furo- and thieno[3,2-c] coumarins derivatives: structure, electronic spectra and TD-DFT study. J. Mol. Str..

[cit13] Ibrahim M. A., Abdel Halim S., Roushdy N., Farag A. A. M., El-Gohary N. M. (2017). Synthesis, DFT band structure calculations, optical and photoelectrical characterizations of the novel 5-hydroxy-4-methoxy-7-oxo-7H-furo[3,2-g] chromene-6-carbonitrile (HMOFCC). Opt. Mater..

[cit14] Roushdy N., Farag A. A. M., Ibrahim M. A., Abdel Halim S., El-Gohary N. M. (2019). Synthesis, spectral characterization, DFT and photosensitivity studies of 1-{[(4-methoxy-5-oxo-5H-furo[3,2-g] chromen-6-yl) methylidene] amino}-4,6-dimethyl-2-oxo-1,2-dihydropyridine-3-carbonitrile (MFCMP). Optik.

[cit15] Farag A. A. M., Ibrahim M. A., Abdel Halim S., Roushdy N., El-Gohary N. M. (2018). Synthesis, DFT calculations, spectroscopic and photovoltaic of the novel N′′, N′′′-bis[(4,9-dimethoxy-5-oxo-5H-furo[3,2-g] chromen-6-yl) methylidene] thiocarbonohydrazide (BFCMT) and its photodiode application. J. Mol. Str*.*.

[cit16] Ibrahim M. A., Abdel Halim S., Roushdy N., Farag A. A. M., El-Gohary N. M. (2018). Synthesis, DFT study and photoelectrical characterizations of the novel 4-methoxyfuro[3,2:6,7]chromeno[2,3-e]benzo[b][1, 4]diazepin-5(12H)-one. Optik.

[cit17] Maridevarmath C. V., Naik L., Negalurmath V. S., Basanagouda M., Malimath G. H. (2019). Synthesis, photophysical, DFT and solvent effect studies on biologically active benzofuran derivative:(5-methyl-benzofuran-3-yl)-acetic acid hydrazide. Chem. Data Collect..

[cit18] Maridevarmath C. V., Naik L., Negalurmath V. S., Basanagouda M., Malimath G. H. (2019). Synthesis, characterization and photophysical studies on novel benzofuran-3-acetic acid hydrazide derivatives by solvatochromic and computational methods. J. Mol. Str..

[cit19] Coskun D., Gunduz B., Coskun M. F. (2019). Synthesis, characterization, and significant optoelectronic parameters of 1-(7-methoxy-1-benzofuran-2-yl) substituted chalcone derivatives. J. Mol. Str..

[cit20] Abdelrazek F. M., Metz P., Kataeva O., Jager A., El-Mahrouky S. F. (2007). Synthesis and molluscicidal activity of new chromene and pyrano[2,3-c] pyrazole derivatives. Arch. Pharm..

[cit21] Lei M., Ma L., Hu L. (2011). A green, efficient, and rapid procedure for the synthesis of 2-amino-3-cyano-1,4,5,6-tetrahydropyrano[3,2-c] quinolin-5-one derivatives catalyzed by ammonium acetate. Tetrahedron Lett.

[cit22] Kumar D., Reddy V. B., Sharad S., Dube U., Suman K. A. (2009). A facile one-pot green synthesis and antibacterial activity of 2-amino-4H-pyrans and 2-amino-5-oxo-5,6,7,8-tetrahydro-4H-chromenes. Eur. J. Med. Chem..

[cit23] Joshi B. D., Srivastava A., Honorato S. B., Tandon P., Pessao O. D. L., Fechine P. B. A., Ayala A. P. (2013). Spectroscopic and quantum chemical study of an alkaloid aristolochic acid I. Spectrochim. Acta, Part A.

[cit24] Xavier R. J., Dinesh P. (2014). Spectroscopic (FTIR, FT-Raman, ^13^C and 1H NMR) investigation, molecular electrostatic potential, polarizability, and first-order hyperpolarizability, FMO and NBO analysis of 1-methyl-2-imidazolethiol. Spectrochim. Acta, Part A.

[cit25] Govindarajan M., Karabacak M. (2012). Spectroscopic properties, NLO, HOMO-LUMO and NBO analysis of 2,5-Lutidine. Spectrochim. Acta, Part A.

[cit26] Nakano M., Fujita H., Takahata M., Yamaguchi K. (2002). Theoretical Study on Second Hyperpolarizabilities of Phenylacetylene Dendrimer: Toward an Understanding of Structure−Property Relation in NLO Responses of Fractal Antenna Dendrimers. J. Am. Chem. Soc..

[cit27] Geskin V. M., Lambert C., Bredas J. L. (2003). Origin of High Second- and Third-Order Nonlinear Optical Response in Ammonio/Borato Diphenylpolyene Zwitterions: The Remarkable Role of Polarized Aromatic Groups. J. Am. Chem. Soc..

[cit28] Mehboob M. Y., Hussain R., Adnan M., Saira A., Farwa U., Irshad Z., Ramzan M., Ashraf Janjua S. (2022). Theoretical modelling of novel indandione-based donor molecules for organic solar cell applications. J. Phys. Chem. Solids.

[cit29] Mehboob M. Y., Zaier R., Hussain R., Adnan M., Asif Iqbal M. M., Irshad Z., Bilal I., Saeed Ashraf Janjua M. R. (2022). In silico modelling of acceptor materials by End-capped and π-linker modifications for high-performance organic solar cells: estimated PCE > 18%. Comput. Theor. Chem..

[cit30] Frisch M. J. , Trucks G. W., Schlegel H. B., Scuseria G. E., Robb M. A., Cheeseman J. R., Scalmani G., Barone V., Mennucci B., Petersson G. A., Nakatsuji H., Caricato M., Li X., Hratchian H. P., Izmaylov A. F., Bloino J., Zheng G., Sonnenberg J. L., Hada M., Ehara M., Toyota K., Fukuda R., Hasegawa J., Ishida M., Nakajima T., Honda Y., KitA O., Nakai H., Vreven T., Montgomery Jr J. A., Peralta J. E., Ogliaro F., Bearpark M., Heyd J. J., Brothers E., Kudin K. N., Staroverov V. N., Kobayashi R., Normand J., Raghavachari K., Rendell A., Burant J. C., Iyengar S. S., Tomasi J., Cossi M., Rega N., Millam J. M., Klene M., Knox J. E., Cross J. B., Bakken V., Adamo C., Jaramillo J., Gomperts R., Stratmann R. E., Yazyev O., Austin A. J., Cammi R., Pomelli C., Ochterski J. W., Martin R. L., Morokuma K., Zakrzewski V. G., Voth G. A., Salvador P., Dannenberg J. J., Dapprich S., Daniels A. D., Farkas O., Foresman J. B., Ortiz J. V., Cioslowski J. and Fox D. J., Gaussian 09 program, Gaussian Inc., Wallingford, CT, 2009

[cit31] Frisch E. , Hratchian H. P., Dennington II R. D., Keith T. A., Millam J., Nielsen A. B., Holder A. J. and Hiscocks J., GaussView Version 5.0.8, Gaussian, Inc., 2009

[cit32] Shahab S., Kumar R., Darroudi M., Borzehandani M. Y. (2015). Molecular structure and spectroscopic investigation of sodium (E)-2-hydroxy-5-((4-sulfonatophenyl) diazenyl) benzoate: a DFT study. J. Mol. Struct..

[cit33] Irfan A., Al-Sehemi A. G., Kalam A. (2013). Structural, electronic and charge transfer studies of dianthra[2,3-b:2,3-f]thieno[3,2-b]thiophene and its analogues: quantum chemical investigations. J. Mol. Struct..

[cit34] Irfan A., Al-Sehemi A. G., Muhammad S. (2014). Investigating the effect of acene-fusion and trifluoroacetyl substitution on the electronic and charge transport properties by density functional theory. Synth. Met..

[cit35] Becke A. D. (1993). Density-functional thermochemistry. III. The role of exact exchange. J. Chem. Phys..

[cit36] Yanai T., Tew D., Handy N. (2004). A new hybrid exchange–correlation functional using the Coulomb-attenuating method (CAM-B3LYP). Chem. Phys. Lett..

[cit37] Lee C. T., Yang W. T., Parr R. G. B. (1988). Development of the Colle-Salvetti correlation-energy formula into a functional of the electron density. Phys. Rev..

[cit38] Petersson D. A., Allaham M. A. (1991). A complete basis set model chemistry. II. Open-shell systems and the total energies of the first-row atoms. J. Chem. Phys..

[cit39] Wolinski K., Hinton J. F., Pulay P. (1990). Efficient implementation of the gauge-independent atomic orbital method for NMR chemical shift calculations. J. Am. Chem. Soc..

[cit40] Sarafran M., Komasa A., Adamska E. B. (2007). Molecular structure, vibrational spectroscopic (FT-IR, FT-Raman), UV and NBO analysis of 2-chlorobenzonitrile by density functional method. J. Mol. Struct..

[cit41] Geerlings P., De Proft F., Langenaeker W. (2003). Conceptual Density Functional Theory. Chem. Rev..

[cit42] Chattaraj K., Giri S. (2007). Stability, Reactivity, and Aromaticity of Compounds of a Multivalent Superatom. J. Phys. Chem. A.

[cit43] Parr R. G., Pearson R. G. (1983). Absolute hardness: companion parameter to absolute electronegativity. J. Am. Chem. Soc..

[cit44] Padmanabhan J., Parthasarathi R., Subramaniaan V., Chattaraj P. K. (2007). Electrophilicity-Based Charge Transfer Descriptor. J. Phys. Chem. A.

[cit45] Kafka S., Pevec A., Proisl K., Kimmel R., Kosmrlj J. (2013). 4-Hydroxy-1-methyl-3-phenylquinolin-2(1H)-one. Acta Cryst., E.

[cit46] Vishnupriya R., Suresh J., Sivakumar S., Kumar R. R., Lakshman P. L. N. (2013). 4-(4-Fluorophenyl)-6-methylamino-5-nitro-2-phenyl-4H-pyran-3-carbonitrile. Acta Cryst., E.

[cit47] Al-Otaibi J. S., Al-Wabli R. I. (2015). Vibrational spectroscopic investigation (FT-IR and FT-Raman) using ab initio (HF) and DFT (B3LYP) calculations of 3-ethoxymethyl-1,4-dihydroquinolin-4-one. Spectrochim. Acta Part A Mol. Biomol. Spectrosc..

[cit48] Li X., Hopmann K. H., Hudecova J., Isaksson J., Novotna J., Stensen W., Andrushchenko V., Urbanova M., Svendsen J. S., Bouř P., Ruud K. (2013). Determination of Absolute Configuration and Conformation of a Cyclic Dipeptide by NMR and Chiral Spectroscopic Methods. J. Phys. Chem. A.

[cit49] Karabacak M. (2009). the spectroscopic (FT-IR and FT-Raman) and theoretical studies of 5-bromo-salicylic acid. J. Mol. Struct..

[cit50] Manohar M. (2008). *et al.*, Review of Particle Physics. Spectrochim. Acta, Part A.

[cit51] Roeges N. P. G. , A Guide to the Complete Interpretation of Infrared Spectra of Organic Structures, John Wiley and Sons Inc., New York, 1994

[cit52] Szafran M., Komasa A., Bartoszak-Adamska E. (2007). Crystal and molecular structure of 4-carboxypiperidinium chloride (4-piperidinecarboxylic acid hydrochloride). J. Mol. Struct..

[cit53] Cheng L. T., Tam W., Stevenson S. H., Meredith G. R., Rikken G., Marder S. R. (1991). Experimental investigations of organic molecular nonlinear optical polarizabilities. 1. Methods and results on benzene and stilbene derivatives. J. Phys. Chem..

[cit54] Karna S. P., Prasad P. N., Dupuis M. (1991). Nonlinear optical properties of p-nitroaniline: An ab initio time-dependent coupled perturbed Hartree-Fock study. J. Chem. Phys..

[cit55] Kaatz P., Donley E. A., Shelton D. P. (1998). A comparison of molecular hyperpolarizabilities from gas and liquid phase measurements. J. Chem. Phys..

[cit56] Nalwa H. S. and Miyata S., Nonlinear Optics of Organic Molecules and Polymers, CRC Press, New York, 1997

[cit57] Sajan D., Josepha L., Vijayan N., Karabacak M. (2011). Natural bond orbital analysis, electronic structure, non-linear properties, and vibrational spectral analysis of l-histidinium bromide monohydrate: a density functional theory. Spectrochim. Acta Part A Mol. Biomol. Spectrosc..

[cit58] Zhang R., Dub B., Sun G., Sun Y. (2010). Experimental and theoretical studies on *o-, m-* and *p*-chlorobenzylideneaminoantipyrines. Spectrochim. Acta Part A Mol. Biomol. Spectro..

[cit59] Chattaraj K., Giri S. (2007). Stability, Reactivity, and Aromaticity of Compounds of a Multivalent Superatom. J. Phys. Chem. A.

